# Different ecological processes determined the alpha and beta components of taxonomic, functional, and phylogenetic diversity for plant communities in dryland regions of Northwest China

**DOI:** 10.7717/peerj.6220

**Published:** 2019-01-10

**Authors:** Jianming Wang, Chen Chen, Jingwen Li, Yiming Feng, Qi Lu

**Affiliations:** 1 College of Forestry, Beijing Forestry University, Beijing, China; 2 Institute of Desertification Studies, Chinese Academy of Forestry, Beijing, China

**Keywords:** Drylands, Facets of diversity, Ecological scale, Biogeography of deserts, Alpha diversity, Beta diversity

## Abstract

Drylands account for more than 30% of China’s terrestrial area, while the ecological drivers of taxonomic (TD), functional (FD) and phylogenetic (PD) diversity in dryland regions have not been explored simultaneously. Therefore, we selected 36 plots of desert and 32 plots of grassland (10 × 10 m) from a typical dryland region of northwest China. We calculated the alpha and beta components of TD, FD and PD for 68 dryland plant communities using Rao quadratic entropy index, which included 233 plant species. Redundancy analyses and variation partitioning analyses were used to explore the relative influence of environmental and spatial factors on the above three facets of diversity, at the alpha and beta scales. We found that soil, climate, topography and spatial structures (principal coordinates of neighbor matrices) were significantly correlated with TD, FD and PD at both alpha and beta scales, implying that these diversity patterns are shaped by contemporary environment and spatial processes together. However, we also found that alpha diversity was predominantly regulated by spatial structure, whereas beta diversity was largely determined by environmental variables. Among environmental factors, TD was most strongly correlated with climatic factors at the alpha scale, while with soil factors at the beta scale. FD was only significantly correlated with soil factors at the alpha scale, but with altitude, soil and climatic factors at the beta scale. In contrast, PD was more strongly correlated with altitude at the alpha scale, but with soil factors at the beta scale. Environment and space explained a smaller portion of variance in PD than in TD and FD. These results provide robust evidence that the ecological drivers of biodiversity differ among different scales and facets of diversity. Future research that focuses on the comparisons among TD, FD and PD would likely provide new insights into elucidating the underlying community assembly.

## Introduction

Understanding the fundamental processes that underlie biogeographic patterns of biodiversity has been a focus of biogeography and ecology (Gaston, 2000; Anderson et al., 2011). In past decades, the biogeography and drivers of the plant diversity across large scales have been extensively investigated (Qian & Ricklefs, 2012; Tang et al., 2012; Chen et al., 2016). Indeed, the majority of theories related to explaining diversity gradients can largely be summarized into two classes: niche and neutral theories. Niche theory emphasizes the importance of contemporary environment, such as abiotic (e.g., climate and soil attributes) and biotic factors (Chase & Leibold, 2003; Tang et al., 2012; Ulrich et al., 2014). It suggests that diversity patterns are largely determined by environmental filtering (Chesson, 2000; Chase & Leibold, 2003). In contrast, the influence of spatial processes was highlighted by neutral theory. It implied that species diversity patterns were mainly regulated by spatial processes (e.g., drift and dispersal limitation; Hubbell, 2001). It is widely reported that both environmental and spatial factors could strongly influence plant diversity, yet no consensus has been reached on the relative contribution of niche and neutral processes to plant diversity across different geographic regions and scales (Steinitz et al., 2006; Legendre et al., 2009).

The arid, semi-arid and dry-subhumid ecosystems (i.e., drylands) of northwest China experience a continual natural vegetation gradient from desert to meadow steppe, occupying more than 30% of the terrestrial area of China. Unfortunately, these dryland ecosystems are expanding and changing in amounts and patterns of precipitation, as a result of desertification and global environmental changes (Reynolds et al., 2007; Dai, 2013). Such changes may have a substantial influence on biodiversity and associated ecosystem functions (Maestre, Salguero-Gómez & Quero, 2012; Vicente-Serrano et al., 2012; Delgado-Baquerizo et al., 2013). Although plant community assembly in these ecosystems has been well documented (Tang et al., 2012; Wang et al., 2017), the previous studies mainly focused on taxonomic diversity (TD), and they rarely concentrated on other diversity facets such as phylogenetic diversity (PD) or functional diversity (FD) (but see Chi, Tang & Fang, 2014).

Indeed, taxon-based approach cannot take into account the difference in evolutionary history and ecological characteristics between species, and thus may generate biased conclusions for the dominant factors underlying community patterns (Swenson et al., 2012; Purschke et al., 2013). Therefore, new biodiversity metrics which can incorporate functional and phylogenetic information have recently been proposed (Cavender-Bares et al., 2009; Swenson, 2013). FD mainly reflects the information of ecological, physiological and morphological traits, whereas PD mainly reflects the accumulated evolutionary history of a community (Webb et al., 2002; Petchey & Gaston, 2006). It is widely thought that both FD and PD may be positively related to TD, because the presence of more species can mean more species traits and lineages (Losos, 2008). However, the difference in evolutionary history and environmental conditions may cause FD and PD of two communities with equal TD to differ significantly (Safi et al., 2011; Tucker & Cadotte, 2013). Notably, the relationship between FD and PD may be strongly influenced by environmental gradients (Blomberg, Garland & Ives, 2003). FD and PD may covary in different ways along spatial scales and environmental gradients (Bernard-Verdier et al., 2013). These inconsistencies may also induce substantial differences between the dominant factors of these diversity facets. Comparing the difference among TD, FD and PD would shed new insights into the underlying drivers of community assembly (Kraft et al., 2007; Cavender-Bares et al., 2009). However, the processes that determine the TD, FD and PD in dryland regions have not been explored simultaneously.

Biological diversity can be characterized by partitioning regional diversity into alpha (within sites) and beta (among sites) diversity (Jost, 2007; Ricotta & Szeidl, 2009). In addition, the relative role of underlying processes may differ remarkably depending on spatial scales, and these ecological processes are usually spatially structured (Tang et al., 2012). For example, it is reported that competition and random dispersal may play dominant roles at the local scale (alpha), while environmental filtering and historical processes may strongly affect beta (regional) diversity (Cornwell, Schwilk & Ackerly, 2006; Cavender-Bares et al., 2009). Therefore, comparing the diversity patterns among different facets at different scales of analysis may be necessary to determine assembly processes. Despite this, to date, the processes that determine the alpha and beta diversity of different facets have not been elucidated synchronously.

To compare the biogeographic patterns and drivers of TD, FD and PD at the alpha and beta levels, we selected 68 sites from a typical dryland region of China. Both alpha and beta components of TD, FD and PD were calculated by applying consistent sampling and analytical methods. Then, we quantified the phylogenetic signals of plant traits to explore the correlations among TD, FD and PD. Specifically, we mainly attempt to address the following three specific questions: (1) Can environmental or spatial factors significantly influence TD, FD and PD at both alpha and beta scales? (2) Do the responses of these diversity facets to the ecological processes differ between the alpha and beta scales? (3) Do the responses of species diversity to these ecological processes differ among three diversity facets?

## Materials and Methods

### Study sites

**Figure 1:**
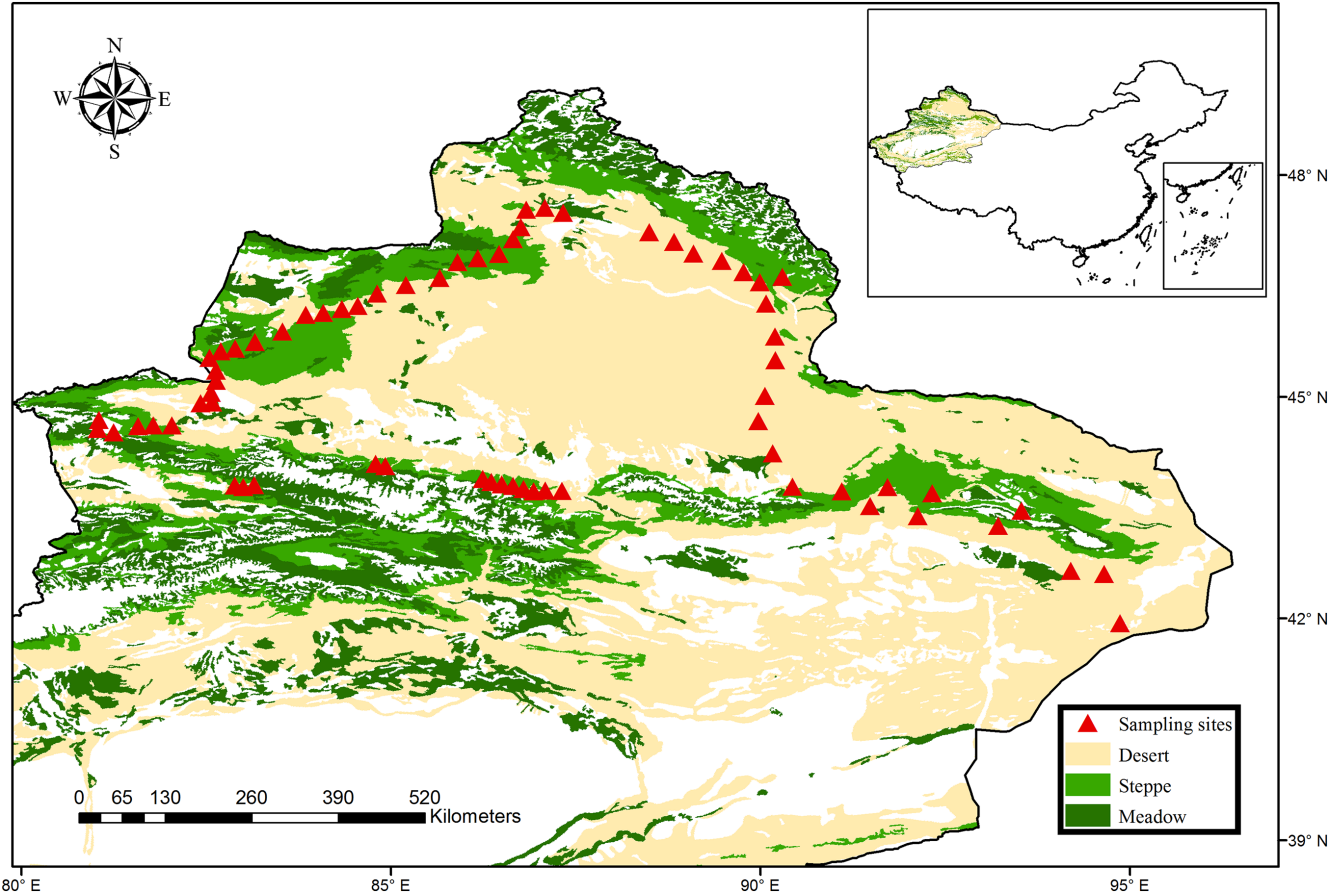
Map of sampling sites across the typical dryland of northwest China. The vegetation data set is provided by Data Center for Resources and Environmental Sciences, Chinese Academy of Sciences (RESDC) (http://www.resdc.cn), and the maps were created using ArcGIS 10 (http://www.esri.com/software/arcgis).

The northern Xinjiang of China, one of the world’s largest dryland regions (including arid, semi-arid and dry-subhumid region), covers more than 450,000 km^2^. The climate is controlled by the continental air mass, changing from arid to semi-arid and dry-subhumid zones. Consequently, four major vegetation types can be identified in this region, including meadow steppe, typical steppe, desert steppe and desert. In 2016, a total of 68 sites with an interval of 10–30 km were sampled from the typical regions of northern Xinjiang, which covered all major climate zones and vegetation types (Fig. 1). Briefly, these sites included three climatic zones (33 sites for arid zones, 35 sites for semi-arid and dry-subhumid zones) and four vegetation types (36 sites for desert, 11 sites for desert steppe and 21 sites for typical steppe and meadow steppe). These sites spanned a broad environmental gradient (the mean annual precipitation (MAP) ranges from 43 to 458 mm; mean annual temperature (MAT) ranges from −0.6 to 9.0 °C) and altitude gradient (altitude ranges from 216 to 2,153 m, with an average of 1,198 m). The dominant species of the grassland were Fabaceae, Asteraceae and Poaceae, while the plant communities in the desert were predominantly dominated by Amaranthaceae and Zygophyllaceae (More species details in Table S1).

### Field sampling

Sampling sites were selected based on the following two criteria: (1) sites should represent the local vegetation; (2) sites should have nearly intact natural plant communities, with no/light animal grazing. At each site, a 10 × 10 m plot was established from the representative vegetation which applied the same plot size with previous studies (Tang et al., 2012; Chi, Tang & Fang, 2014; Li et al., 2016). Meanwhile, the geographic factors (latitude and longitude) were recorded with a GPS (GPSMAP; Garmin, Olathe, KS, USA), and then we also measured topographic factors (altitude and slope). After that, all vascular plant species occurrence was recorded and then summarized at the site level. Finally, 15 soil samples (10 cm in depth) were randomly collected from each plot, and then the 15 soil samples were combined to obtain one composite sample. Then these composite samples were stored in thermal insulated boxes (at 4 °C) for determining the soil attributes.

### Environmental data

Soil attributes, including soil pH (pH), soil total phosphorus and nitrogen (TSN and TSP), total organic carbon (TOC), available nitrogen (AN) and moisture content (SM), soil N:P and C:N ratios were used in this study. The methods and details for determining these soil attributes have been described by our previous studies (Wang et al., 2017).

We selected a range of climatic variables, including MAP, actual evapotranspiration (AET), MAT, mean temperature of the coldest month (MTCM), mean temperature of the warmest month (MTWM), and potential evapotranspiration (PET). MAP, MAT, MTCM and MTWM data were extracted from the WorldClim global climate database (http://www.worldclim.org), and AET and PET data were derived from CGIAR-CSI (http://www.cgiar-csi.org). All data for the study sites were extracted using the geographical coordinates at a resolution of 1 × 1 km.

To reduce soil attributes and climate data redundancy, principal components analysis (PCA) was applied using the redundancy analyses (RDA) command within vegan package (Oksanen et al., 2016). Together, the first two climate PCs and first four soil PCs which explained more than 90% of the total variation were used in the following analysis.

### Phylogenetic and species trait data

Firstly, all plant species were identified based on Angiosperm Phylogeny Group III system using the Plant List database (http://www.theplantlist.org/). Together, a total of 233 plant species were identified from the overall list. After that, the completed phylogenetic tree with branch lengths was directly assembled within the plant phylogeny software “Phylomatic 3.0” (http://phylodiversity.net/phylomatic/; Webb & Donoghue, 2005), based on the ultrametric phylogeny of Zanne et al. (2014). After that, the phylogenetic tree was randomly solved by “multi2di” function, and then we ultrametrized the tree using “compute.brlen” function from ape package. The phylogenetic tree of our study is available in the Fig. S1.

We selected nine plant traits for all species: growth form, plant height, leaf texture, leaf shape, fruit type, fruit ripening period, length of the flowering period, flowering onset, life history. All plant traits were compiled or derived from online databases of the Flora of China (http://foc.eflora.cn/). These plant traits were thought to characterize different dimensions of the plant’s functional niche with respect to species morphology, life-history and phenology (Cornelissen et al., 2003; Litchman & Klausmeier, 2008; Grime et al., 2008; Moles et al., 2009). Qualitative data (e.g., growth form, leaf texture, leaf shape, fruit type, life history) were re-coded as a quantitative variable (Table S2). PCA was performed on the standardized trait value to avoid trait data redundancy (Purschke et al., 2013; Arnan, Cerdá & Retana, 2015) using the RDA command within vegan package (Oksanen et al., 2016). After that, the resulting PCA axes were used to construct the Euclidean trait distance matrix.

Phylogenetic signal, the tendency for related species to resemble each other, could be used to explain the relationship between FD and PD (Blomberg, Garland & Ives, 2003). Significant phylogenetic signals mean the functional similarity of closely related species, and the similarity patterns between FD and PD (Cavender-Bares et al., 2009). Hence, we tested the presence of phylogenetic signals of each plant trait using Blomberg’s *K* (Blomberg, Garland & Ives, 2003) and Pagel’s λ test (Pagel, 1999). Blomberg’s *K* > 1 means the stronger phylogenetic signal than expected by Brownian motion (BM), whereas *K* < 1 indicates the weaker phylogenetic signal than expected by BM. Similarly, a higher Pagel’s λ value means a stronger phylogenetic signal. Furthermore, the significance of the *K* values was tested through comparing to null distributions by shuffling species labels at the tip of the phylogeny 10,000 permutations; while the statistical significance of λ values was examined through a likelihood ratio test.

### Partitioning diversity

The additive partitioning of Rao’s quadratic entropy was used to separate diversity within and among communities, into alpha, beta and gamma components (Hardy & Senterre, 2007; Arnan, Cerdá & Retana, 2015). Being distance-based, it provides a flexible and standardized methodology for comparing and partitioning different facets of diversity (e.g., TD, FD and PD) among species (De Bello et al., 2010; Devictor et al., 2010; Bernard-Verdier et al., 2013). Furthermore, Rao’s estimates of FD and PD are relatively independent of TD (Mouchet et al., 2010). Within each community *k*, alpha diversity was estimated using Rao’s coefficient of diversity (Rao, 1982; Pavoine, Dufuor & Chessel, 2004) modified for presence-absence data.

$${\rm{\alpha Rao}}(k) = \sum\limits_{i = 1}^n {\sum\limits_{j = 1}^n {dij} } $$

Where αRao(*k*) is the alpha diversity within in community *k*; and *d_ij_*, is the distance between species *i* and *j*, which can be taxonomic, functional or phylogenetic.

The β-diversity was defined as the variation in species composition among different sites (Whittaker, 1960), whereas this concept has recently been extended to describe phylogenetic and functional dissimilarity among communities (Graham & Fine, 2008). Rao’s dissimilarity index (Rao, 1982) was used to calculate taxonomic, functional and phylogenetic β-diversity among communities, and that is the expected distance (e.g., taxonomic, phylogenetic and functional distance) between two individuals selected from two distinct communities randomly.

$${\rm{\beta Ra}}{{\rm{o}}_{{\rm{pairwise}}}}(k,{\rm{l}}) = ({\rm\gamma} (k{\rm{ + 1}}){\rm{ - }}\overline {\rm \alpha} (k,{\rm{l}})){\rm{/}}{\rm \gamma} (k{\rm{ + 1}})$$

Where γ_(*k*+1)_ is the gamma diversity of the pair of communities, whereas ${\rm{\overline\alpha}}(k,{\rm{l}})$ is the mean α-diversity of the two communities. To accurately quantify β-diversity independently of α-diversity, we applied Jost’s correction (Jost, 2007) to γ and α diversity, prior to calculations (De Bello et al., 2010). All above calculations were conducted using function “rao” (De Bello et al., 2010) in R package.

Different distance measures were used to estimate the Rao quadratic entropy index, depending on the facet of diversity considered. Taxonomic distances between species were measured as *d_ij_* = 1 when *i* ≠ *j*, and *d_ij_* = 0 when *i* = *j*. To compute functional distances between species, the resulting PCA axes of traits were used to calculate Euclidean distances. Finally, the cophenetic distances from the phylogenetic tree were used to measure the phylogenetic distances between species.

### Statistical analyses

To reduce the autocorrelation between environmental variables and spatial distance, the analysis of principal coordinates of neighbor matrices (PCNM) based on geographical coordinates was used to obtain the spatial variables (Dray, Legendre & Peres-Neto, 2006). Specially, total 19 PCNM vectors with positive eigenvalues were regarded as explanatory variables. Then the spatial autocorrelation between the alpha and beta components of TD, PD and FD was examined by Mantel tests and Moran’s *I* (Oksanen et al., 2016).

Second, RDA were used to explore the relationships among alpha and beta components of three diversity facets, climatic, soil, topographic and spatial variables. Since dissimilarity matrix cannot be used directly in redundancy-analysis framework, the scores of the significant axes of the principal coordinate analysis based on the Rao’s dissimilarity matrix represented the value of each diversity facets. To prevent data overfitting, all variables were subjected to forward-selection until *P* < 0.05 within the “packfor” package (Dray, Legendre & Blanchet, 2009). When more than one variable was retained in the final model, the independent contribution of each retained variable would be assessed. Variation partitioning analyses were conducted to further determine the relative influence of environmental and spatial factors on alpha and beta components of three diversity facets within the vegan package (Oksanen et al., 2016).

## Results

### Phylogenetic signals

Among nine functional traits, only growth form, leaf texture, fruit type, fruit ripening period and flowering onset showed significant phylogenetic signals in both Blomberg’s *K* and Pagel’s λ test, whereas plant height and leaf shape did not (Table 1). Nevertheless, the Pagel’s λ and Blomberg’s *K* values for each functional trait were less than 1, implying weak phylogenetic signals. These results suggest that evolutionary history or phylogenetic relationships may only significantly influence a part of functional traits types, and PD could not be used as a simple proxy for TD in dryland regions of China. Furthermore, we found that the length of flowering period and life history had a significant phylogenetic signals according to Pagel’s λ test, but did not in Blomberg’s *K* test. Indeed, both statistic values of above two traits were a bit lower than other traits that showed significant signals of Blomberg’s *K* and Pagel’s λ test. This inconsistency between *P*-value of the Pagel’s λ and Blomberg’s *K* test may be caused by the difference in methods of computing significance of signals.

**Table 1:** Phylogenetic signals of plant functional traits in the typical dryland of northwest China.

Plant trait	Blomberg’s *K*		Pagel’s λ	
	*K*	*P*	λ	*P*
Growth form	0.153	0.0002	0.893	<0.0001
Plant height	0.043	0.1650	0.763	0.115
Leaf texture	0.229	0.0001	0.781	<0.0001
Leaf shape	0.039	0.0630	0.294	0.083
Fruit type	0.979	0.0010	0.999	<0.0001
Fruit ripening period	0.044	0.0360	0.782	<0.0001
Length of the flowering period	0.041	0.0940	0.519	<0.0001
Life history	0.033	0.2080	0.353	0.024
Flowering onset	0.054	0.0034	0.658	<0.0001

### Taxonomic, functional and phylogenetic diversity

**Figure 2:**
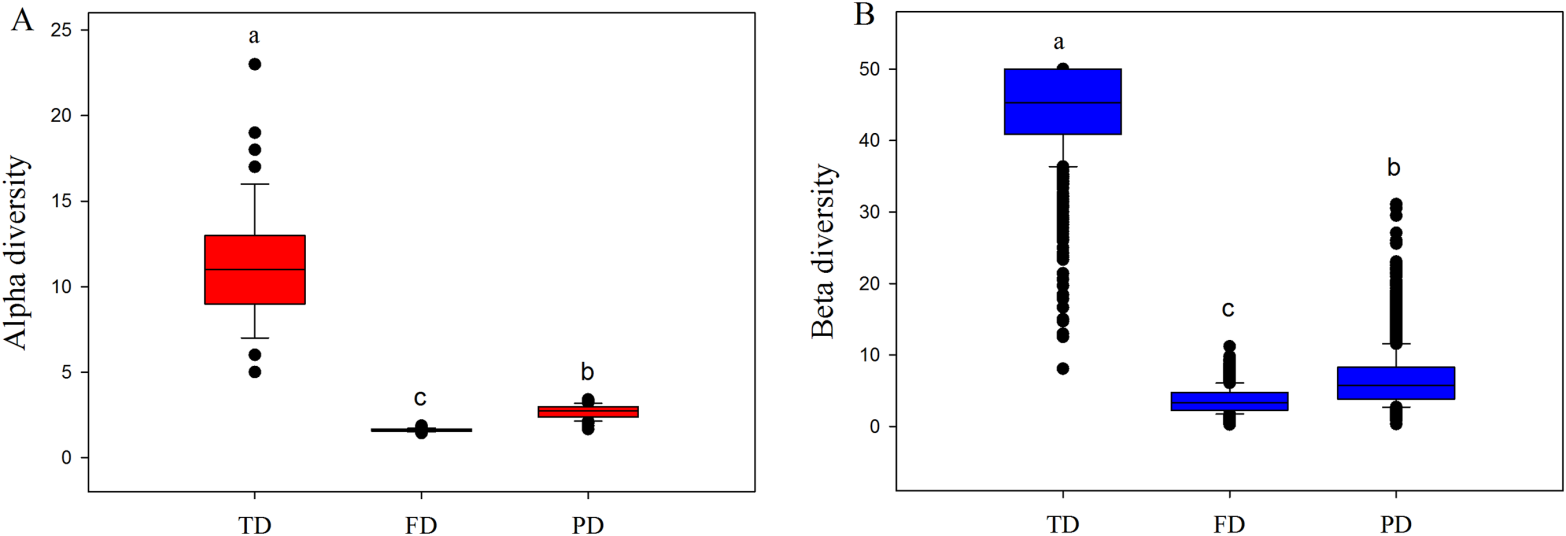
Boxplots of alpha (A) and beta (B) components of taxonomic (TD), functional (FD) and phylogenetic (PD) diversity. Letters indicate significant differences, *P* < 0.05.

Both alpha and beta components of diversity differed significantly among different facets, where the values of these components were highest in TD, intermediate in PD, and lowest in FD (Fig. 2). This may be explained by the following two reasons. First, high drought and temperature of drylands may generate intense environmental stress, and such pressure may cause more closely related species with similar functions to enter into the community (Wake, 1991; Webb et al., 2002). For example, our 233 species only belonged to 39 families and 134 genera, and were classified into simpler functional traits types (Table S2). Therefore, plant communities would have low FD and PD, at a given level of TD. Furthermore, convergent evolution may cause species with different or far evolutionary relationships to have similar functional traits to adapt to the harsh environment conditions (Wake, 1991). This may explain why the values of FD were obviously lowest among three diversity facets.

**Figure 3:**
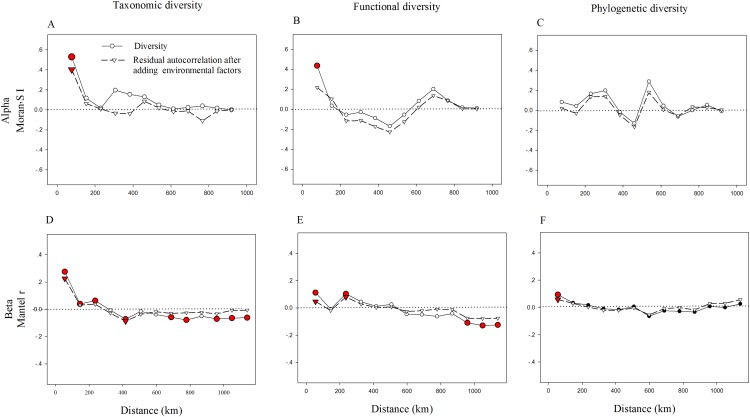
Correlograms of spatial autocorrelation of taxonomic (A, D), functional (B, E), phylogenetic (C, F) diversity and residual autocorrelation after adding environmental factors into the models. We used the Moran’s I and Mantel tests for alpha (A–C) and beta (D–F) components, respectively. Red circles (squares or triangles) indicate significant values (*P* < 0.05), while open circles (squares or triangles) denote non-significant values.

We also found strong spatial autocorrelations among beta components of diversity in the above three facets (Fig. 3). Notably, the significant spatial autocorrelations among alpha components of diversity were found in TD and FD, while not in PD (Fig. 3). This demonstrates that in the relationships among spatial factors, the alpha components of TD and FD are spatially structured, while not with the alpha components of PD. Adding environmental variables into the models could significantly reduce spatial autocorrelation, especially for alpha components of FD and beta components of TD and FD (Fig. 3). It may imply that environmental factors may be an important cause of spatial autocorrelation in these diversity facets. Furthermore, the correlation coefficient of these diversity facets went up and down with increasing distance. These patterns may be partly caused by the spatial structure of environmental variables, since environmental variables also showed similar spatial autocorrelations (Fig. S2).

### The influence of soil, climate, topography and space on the taxonomic, functional and phylogenetic diversity

Stepwise multiple regressions analysis showed that the alpha components of TD was mainly predicted by PC1_soil_, PC1_clim_ and PCNMs (*R*^2^ = 0.156, 0.183 and 0.283, respectively; *P* < 0.05, Table 2), whereas the alpha components of FD was mainly predicted by PC4_soil_ and PCNMs (*R*^2^ = 0.064 and 0.413, respectively; *P* < 0.05, Table 2). The alpha components of PD was primarily explained by altitude and PCNMs (*R*^2^ = 0.117 and 0.213, *P* < 0.05, respectively; Table 2).

**Table 2:** Explanatory variables selected from forward-selected procedure in RDA for explaining the alpha component of the taxonomic (TD), functional (FD) and phylogenetic (PD) diversity.

	Variables	Individual contribution of variable (%)	*P*	Model *R*_adj_^2^	Model *P*
Alpha TD	PC1_soil_	15.88	<0.0001	0.595	<0.0001
	PC2_clim_	18.33	<0.0001		
	PCNM3	19.58	<0.0001		
	PCNM12	3.23	<0.05		
	PCNM16	5.47	<0.05		
Alpha FD	PC4_soil_	6.38	<0.001	0.443	<0.0001
	PCNM2	4.95	<0.05		
	PCNM3	5.15	<0.05		
	PCNM9	31.21	<0.0001		
Alpha PD	Altitude	11.71	<0.001	0.287	<0.0001
	PCNM3	6.49	<0.001		
	PCNM7	8.83	<0.001		
	PCNM15	5.93	<0.001		

**Note:**

RDA, redundancy analysis; PCNM, principal coordinates of neighbor matrices; PC, Soil and climate principal component.

We found that the beta components of three facets of diversity were significantly predicted by different combinations of soil, climate, topography and space (Table 3). In beta-level models, FD was significantly predicted by soil, climate, topography and space (PCNMs) together. TD was significantly predicted by soil, climate and space (PCNMs) together. However, PD could only be significantly predicted by soil and space (PCNMs) together.

**Table 3:** Explanatory variables retained in the forward-selected models for explaining beta diversity for the taxonomic (TD), functional (FD) and phylogenetic (PD) diversity.

Variables		Beta TD		Beta FD		Beta PD	
		*R*_adj_^2^ Cum	*P*	*R*_adj_^2^ Cum	*P*	*R*_adj_^2^ Cum	*P*
Environment	Altitude			0.466	<0.0001		
	PC1_soil_	0.569	<0.0001	0.579	<0.0001	0.291	<0.0001
	PC1_clim_	0.321	<0.0001	0.491	<0.0001		
	PC2_clim_	0.245	<0.0001				
Space	PCNM1					0.175	<0.01
	PCNM3	0.318	<0.0001				
	PCNM8			0.249	<0.0001		
	PCNM7	0.132	<0.05			0.134	<0.05
	PCNM13	0.158	<0.01				

**Note:**

PCNM, principal coordinates of neighbor matrices. PC, Soil and climate principal component *R*_adj_^2^ Cum, adjusted cumulative square of the sum of all canonical eigenvalues (expressing explained variance).

### The relative influence of spatial and environmental factors on the taxonomic, functional and phylogenetic diversity

**Figure 4:**
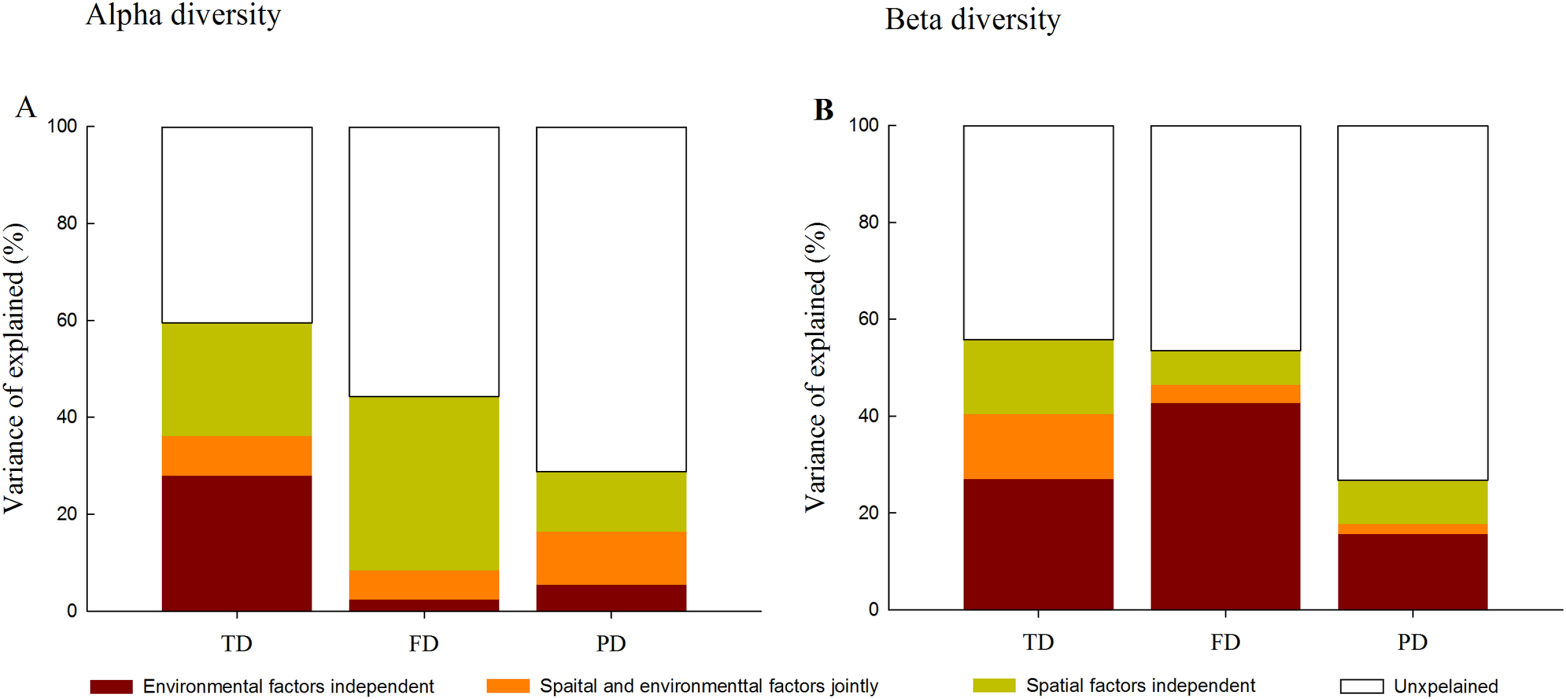
Variation partitioning for the relative influence of environmental and spatial factors on the alpha (A) and beta (B) components of taxonomic (TD), functional (FD) and phylogenetic (PD) diversity. Notes: environmental factors independent, individual influence of environmental factors; spatial and environmental factors jointly, spared influence of spatial and environmental factors; spatial factors independent, individual influence of spatial factors; unexplained, the unexplained variation.

Variation partitioning analysis further quantified the relative influence of environment and space on different facets of diversity. At the alpha level, spatial and environmental factors together explained 59.5%, 44.4% and 28.7% of the total variance in TD, FD and PD, respectively (Fig. 4). Compared with environmental factors which individually explained 2.4% and 5.4% of the total variance in FD and PD, spatial factors individually explained a larger portion of the variation (35.9% and 12.3%). Furthermore, spatially structured environmental factors explained 8.2%, 6.0% and 11.0% of the total variance in TD, FD and PD, respectively.

In contrast, at the beta level, environmental variables individually explained a larger fraction of the total variation in three facets of diversity (27.0%, 42.7% and 15.7%) than spatial factors (15.4%, 7.1% and 9.1%; Fig. 4). Among different facets of diversity, pure environmental variables were the best predictors for TD, FD and PD, whereas pure environmental variables explained more variation in FD than that in TD and PD. Furthermore, when FD was calculated through individual functional traits, we found that the response of FD to environmental and spatial factors varied among different functional traits types (Fig. S3).

## Discussion

Indeed, the relative influence of niche and neutral processes on plant diversity has long been controversial (Jones et al., 2008; Myers et al., 2013; Liu, Tang & Fang, 2015; Chen et al., 2016; Murphy, Salpeter & Comita, 2016). It has been reported recently that both contemporary environment and space may have a great effect on plant diversity; nevertheless, their relative influences vary across study scales and habitat types (Blundo, González-Espinosa & Malizia, 2016; Liu, Tang & Fang, 2015). Our study found that environment and pure spatial factors could significantly influence TD, FD and PD at both alpha and beta scales, implying that contemporary environment and spatial processes are two important drivers for these diversity facets (Blundo, González-Espinosa & Malizia, 2016; Liu, Tang & Fang, 2015). In fact, such similar evidence also has been found in some previous studies in the grassland or desert of China (Tang et al., 2012; Chi, Tang & Fang, 2014; Wang et al., 2017). This provides robust evidence indicates that niche and neutral processes are not mutually exclusive, but work together to determine species coexistence and diversity in dryland regions of China (Gravel et al., 2006; Chase, 2007; Legendre et al., 2009). Notably, a large fraction of the variance in three diversity facets still remained unexplained at both alpha and beta scales, suggesting that other ecological processes and/or non-measured ecological variables may also play important roles in shaping these diversity patterns (Legendre et al., 2009; Myers et al., 2013; Chi, Tang & Fang, 2014).

Among environmental factors, climate, especially water availability has been cited as one of the most important environmental factors that control plant diversity in dryland regions (Tang et al., 2012; Ulrich et al., 2014). Our study found that although climate affected alpha TD more strongly, soil factors had a more important influence on three facets of diversity at the beta scale. In addition, soil factors and altitude were the best predictors for alpha FD and alpha PD, respectively. These results suggest that local factors (e.g., soil conditions and topography) may play a more important role than climate in controlling these diversity facets through diverse processes, such as recruitment limitation (Grubb, 1977) or resource competition (Stevens & Carson, 2002). There are several interpretations for the results presented here. First, the strong covariation between climate and these local factors (Table S1) makes it difficult to quantify their pure influence precisely (Gaston, 2000; Gilbert & Lechowicz, 2004). Second, climate plays a fundamental role in ecosystem nitrogen cycling in the dryland of China (Fernandez-Going et al., 2013; Wang et al., 2014), and thus climate may indirectly regulate these diversity facets through influencing the availability of water and nutrients (Ruiz-Sinoga & Diaz, 2010). Third, in dryland regions, soil water and nutrients resources may be largely redistributed by the rugged and discrete topography (e.g., water retention), and it may lead to fragmentation and patchy distribution of plant communities. This, in turn, would further accentuate the spatial heterogeneity of water and nutrients supply (Tongway & Ludwig, 2005; Reisner et al., 2013). For example, we observed that soil heterogeneity may be significantly higher than climate, since the mean euclidean distance of soil attributes (with an average of 2.58 ± 0.03(SE)) was significantly higher than that of climate (with an average of 1.75 ± 0.02(SE)). Such higher heterogeneous microhabitats and soil conditions may provide more chances for plant species to adapt to suitable habitats, therefore soil attributes or topography may be more important. In addition, our results are inconsistent with the viewpoints of previous studies at larger scales (Qian & Ricklefs, 2012; Ulrich et al., 2014), but supported by the study in the grassland of Xinjiang (Chi, Tang & Fang, 2014). This may indicate that the relative influence of climate and local factors on plant diversity may be scale-dependent (Legendre et al., 2009).

In agreement with Bernard-Verdier et al. (2013), our results showed that three facets of diversity differed in the response to environmental and spatial factors. First, the unexplained variance in PD was clearly larger than that in TD and FD, and both environment and space could only explain a relatively small portion of variance in PD. This may imply that local stochasticity which arises from ecological drifts, unmeasured environmental and spatial factors may have a stronger influence on PD than TD and FD (Myers et al., 2013; Chi, Tang & Fang, 2014). Second, the influence of environmental factors on TD was stronger than that on FD and PD, this may explain why the level of TD was higher than that of FD and PD. Third, the various environmental factors filtered different diversity facets differentially. For example, the alpha components of FD and PD were mainly explained by soil or altitude, whereas the alpha components of TD were more strongly related to climatic factors. Notably, not all functional traits showed significant phylogenetic signals, and the spatial autocorrelation differed obviously among alpha- and- beta-level TD, FD and PD. Our single-trait analyses also suggested that the influence of different processes on FD varied among different functional traits. Arnan, Cerdá & Retana (2015) thought that the degree of niche conservatism might differ across different environmental gradients, and these differences could cause the patterns of three facets of diversity to vary in some gradients. Perhaps these findings could explain why TD, FD and PD showed different responses to ecological processes. Taken together, we emphasize that the comparisons among TD, FD and PD are essential for exploring underlying community assembly (Purschke et al., 2013).

As expected, we also found that the relative contribution of environment and space differed obviously between the alpha and beta scales. Alpha diversity was predominantly regulated by spatial factors (but except TD), whereas beta diversity was largely determined by environmental factors. Given that pure spatial contribution may reflect the influence of dispersal limitation, historical processes, biotic processes and unmeasured underlying environment (Legendre et al., 2009; Smith & Lundholm, 2010). This may suggest that dispersal limitation, biotic and unmeasured factors play more important roles in shaping diversity at the local scale, whereas environmental filtering is more powerful at the regional scale (Cornwell, Schwilk & Ackerly, 2006; Cavender-Bares et al., 2009).

Furthermore, environmental factors explained more beta-level variance in FD and PD than alpha-level variance. It may be partly caused by the ecological or functional difference among different species along environmental gradients. For example, the dominant species in the grassland are mainly herbaceous species (e.g., *Iris tectorum*, *Seriphidium terrae-albae*, *Stipa caucasica*), while plant communities in the desert are predominantly dominated by woody species, such as *Haloxylon ammodendron* and *Krascheninnikovia ceratoides*. Compared with herb species, long-distance dispersal may be more limited for woody species, due to the larger size of individuals and seeds (Allen et al., 2006; Farjalla et al., 2012). In contrast, herb species may be more sensitive to environmental stress, such as drought, than shrub species, due to the more shallow rooting depth and lack of secondary tissue (Ricklefs & Latham, 1992; Costa, Magnusson & Luizao, 2005). When habitat types change from desert to grassland, some herbaceous species might enter the new plant communities, but most shrubs might not. This may cause beta diversity (species turnover) to change more obviously than alpha diversity. Therefore, environmental divergence has weaker effects on alpha diversity than on beta diversity. Another one probable reason is that some spatially structured biotic and abiotic variables which powerfully influence alpha diversity may be missed by our study. Taken together, we highlight that different ecological processes shape the alpha and beta diversity.

## Conclusions

Our study represents an attempt to explore the associations among determinants of TD, FD and PD at the alpha and beta scales, in a typical dryland region of northwest China. We found that environmental and spatial factors were correlated with TD, FD and PD at both alpha and beta scales, implying that these diversity patterns are determined by environmental filtering and spatial processes together. However, we also found that the relative contribution of environment and space differed observably between the alpha and beta scales. This suggests that the ecological processes shaping biodiversity patterns differ remarkably among spatial scales. Furthermore, TD, FD and PD were controlled by various combinations of soil, climate, topography and spatial factors at the alpha and beta scales. Environment and space explained a smaller portion of variance in PD than in TD and FD. From these results, we highlight that the ecological drivers of biodiversity may differ among different facets.

## Supplemental Information

10.7717/peerj.6220/supp-1 Supplemental Information 1 Raw data of three facets of diversity facets and environmental variables in this study.

10.7717/peerj.6220/supp-2 Supplemental Information 2 Functional traits used to calculate the functional diversity included in this study.

10.7717/peerj.6220/supp-3 Supplemental Information 3 Functional traits used to determine the functional diversity and its quantified value.

10.7717/peerj.6220/supp-4 Supplemental Information 4 The correlation matrix between environmental factors.

10.7717/peerj.6220/supp-5 Supplemental Information 5 Phylogenetic tree.

10.7717/peerj.6220/supp-6 Supplemental Information 6 Variance partitioning of the alpha (A) and beta (B) components of functional diversity for each individual functional trait. Notes: environmental factors independent, individual influence of environmental factors; spatial and environmental factors jointly, spared influence of spatial and environmental factors; spatial factors independent, individual influence of spatial factors; unexplained, the unexplained variation.

10.7717/peerj.6220/supp-7 Supplemental Information 7 Correlograms of the taxonomic (TD), functional (FD) and phylogenetic (PD) diversity and residuals after sequentially adding environmental variables into the models.

## Additional Information and Declarations

### Data Availability

The following information was supplied regarding data availability: The raw data is available in the Supplementary Files.
