# Supplementary material for: Different ecological processes determined the alpha and beta components of taxonomic, functional, and phylogenetic diversity for plant communities in dryland regions of Northwest China
Source: PeerJ. 2019 Jan 10;6:e6220. doi: 10.7717/peerj.6220 (PMC6330206; doi:10.7717/peerj.6220)
Supplement: Supplemental Information 2 [file peerj-07-6220-s002.doc]

| **Vegetation types** | **Sites numbers** | **Species numbers** | **Altitude range** | **Dominant and common species** | **Growth-form** | **Life-history** |
| --- | --- | --- | --- | --- | --- | --- |
| Meadow steppe | 3 | 20 | 2101-2153 | *Iris tectorum*  *Phlomoidespratensis*  *Trifolium repens* etc | Herb | Annual  Biennial  Perennial |
| Typical steppe | 18 | 138 | 1059-1958 | *Stipa caucasica,*  *Stipa purpurea,*  *Poa annua*  *Stipa splendens* etc | Herb  Small-subshrub  Subshrub  Dwarf shrub  Semi-shrub  Undershrub  Shrub | Annual  Biennial  Perennial |
| Desert steppe | 11 | 81 | 824-1604 | *Stipa caucasica*  *Stipa purpurea*  *Seriphidium terrae-albae*  *Ceratocarpus arenarius* etc | Herb  Liana  Small-subshrub  Subshrub  Dwarf shrub  Semi-shrub  Undershrub  Shrub | Annual  Biennial  Perennial |
| Desert | 36 | 117 | 216-1723 | *Haloxylon ammodendron*  *Krascheninnikovia ceratoides*  *Seriphidium_terrae-albae* etc | Herb  Liana  Small-subshrub  Subshrub  Dwarf shrub  Semi-shrub  Undershrub  Shrub | Annual  Biennial  Perennial |
