# Supplementary material for: Different ecological processes determined the alpha and beta components of taxonomic, functional, and phylogenetic diversity for plant communities in dryland regions of Northwest China
Source: PeerJ. 2019 Jan 10;6:e6220. doi: 10.7717/peerj.6220 (PMC6330206; doi:10.7717/peerj.6220)
Supplement: Supplemental Information 3 [file peerj-07-6220-s003.doc]

| Trait | Data Type | Trait Types | States |
| --- | --- | --- | --- |
| Growth-Form | Ordinal | Herb | 0 |
|  |  | Liana | 0.125 |
|  |  | Small-Subshrub | 0.25 |
|  |  | Subshrub | 0.375 |
|  |  | Dwarf Shrub | 0.5 |
|  |  | Semi-Shrub | 0.625 |
|  |  | Undershrub | 0.75 |
|  |  | Shrub | 1 |
| Plant-Height | Continuous |  |  |
| Leaf-Texture | Ordinal | Fleshy | 0 |
|  |  | Membranous | 0.25 |
|  |  | Herbaceous | 0.5 |
|  |  | Paper-Quality | 0.75 |
|  |  | Leather-Substance | 1 |
| Leaf Shape | Ordinal | Aciculiform | 0 |
|  |  | Lanceolate | 0.047619 |
|  |  | Pentagon | 0.095238 |
|  |  | Oblong | 0.142857 |
|  |  | Ovate | 0.190476 |
|  |  | Roundness | 0.238095 |
|  |  | Strip-Type | 0.285714 |
|  |  | Cylindrical | 0.333333 |
|  |  | Obovate | 0.380952 |
|  |  | Linear | 0.428571 |
|  |  | Triangle | 0.47619 |
|  |  | Ellipse | 0.52381 |
|  |  | Long-Roundness | 0.571429 |
|  |  | Broadly-Ovate | 0.619048 |
|  |  | Oval | 0.666667 |
|  |  | Oblanceolate | 0.714286 |
|  |  | Subulate | 0.761905 |
|  |  | Hastate | 0.809524 |
|  |  | Clavate | 0.857143 |
|  |  | Cochlear | 0.904762 |
|  |  | Short-Cylindrical | 0.952381 |
|  |  | Semicircular-Cylinder | 1 |
| Fruit-Type | Ordinal | NA | 0 |
|  |  | Capsule | 0.090909 |
|  |  | Achene | 0.181818 |
|  |  | Utricle | 0.272727 |
|  |  | Caryopsis | 0.363636 |
|  |  | Nuts | 0.454545 |
|  |  | Legume | 0.545455 |
|  |  | Drupe | 0.636364 |
|  |  | Schizocarp | 0.727273 |
|  |  | Silique | 0.818182 |
|  |  | Silicle | 0.909091 |
|  |  | Berry | 1 |
| Fruit-Ripening-Period | Continuous |  |  |
| Length-Of-The-Flowering Period | Continuous |  |  |
| Flowering-Onset | Continuous |  |  |
| Life-History | Ordinal | Annual | 0 |
|  |  | Biennial | 0.5 |
|  |  | Perennial | 1 |

Notes: NA, no record
