# Supplementary material for: Different ecological processes determined the alpha and beta components of taxonomic, functional, and phylogenetic diversity for plant communities in dryland regions of Northwest China
Source: PeerJ. 2019 Jan 10;6:e6220. doi: 10.7717/peerj.6220 (PMC6330206; doi:10.7717/peerj.6220)
Supplement: Supplemental Information 4 [file peerj-07-6220-s004.doc]

|  | ATL | Slope | PC1_soil_ | PC2_soil_ | PC3_soil_ | PC4_soil_ | PC1_clim_ |
| --- | --- | --- | --- | --- | --- | --- | --- |
| ATL | .307* |  |  |  |  |  |  |
| Slope | -.538** | -.370** |  |  |  |  |  |
| PC1_soil_ | .263* | .314** | 0 |  |  |  |  |
| PC2_soil_ | -0.153 | -0.116 | 0 | 0 |  |  |  |
| PC3_soil_ | -0.173 | .256* | 0 | 0 | 0 |  |  |
| PC4_soil_ | .638** | 0.182 | -.648** | -0.163 | -0.06 | 0.005 |  |
| PC1_clim_ | -0.042 | -0.215 | 0.166 | -0.146 | -.267* | -0.208 | 0 |
