# Supplementary figures and images for: Different ecological processes determined the alpha and beta components of taxonomic, functional, and phylogenetic diversity for plant communities in dryland regions of Northwest China
Source: PeerJ. 2019 Jan 10;6:e6220. doi: 10.7717/peerj.6220 (PMC6330206; doi:10.7717/peerj.6220)

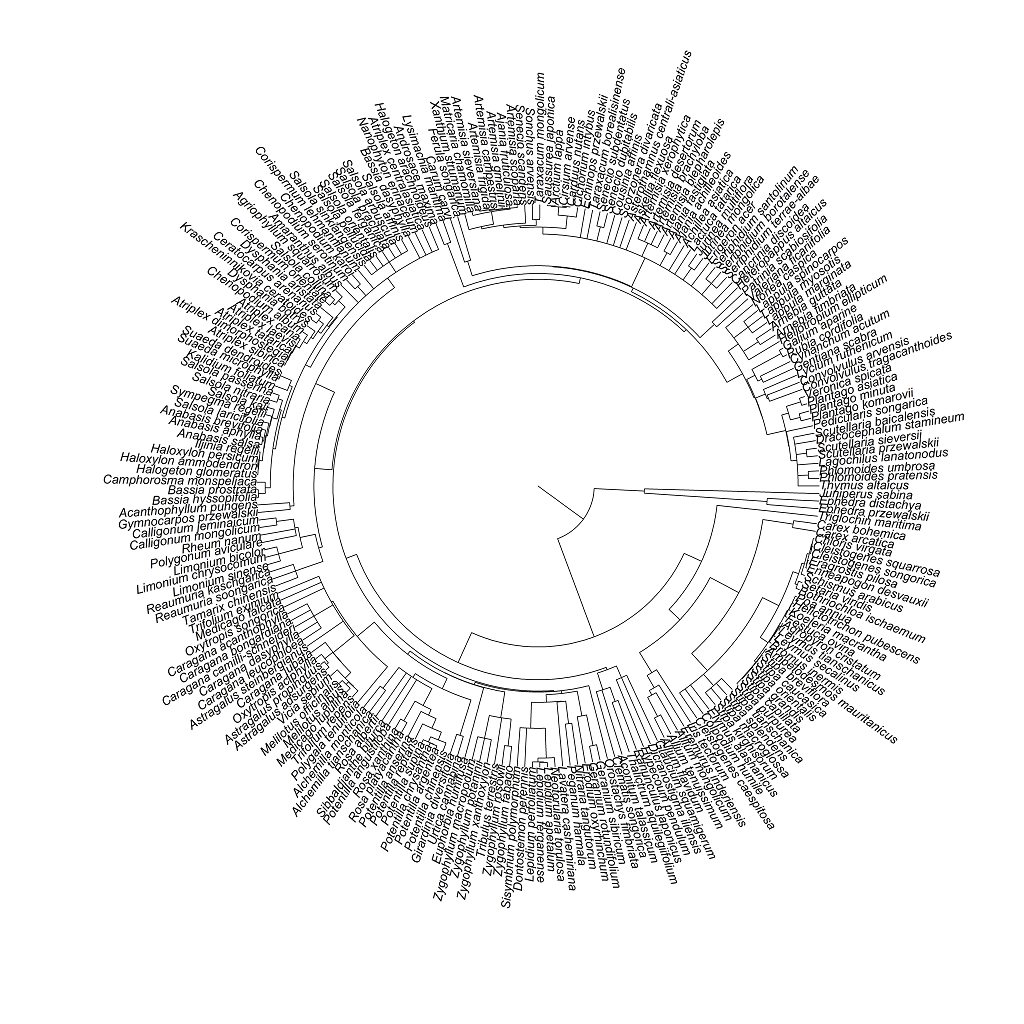

Supplement: Supplemental Information 5 [file peerj-07-6220-s005.png]

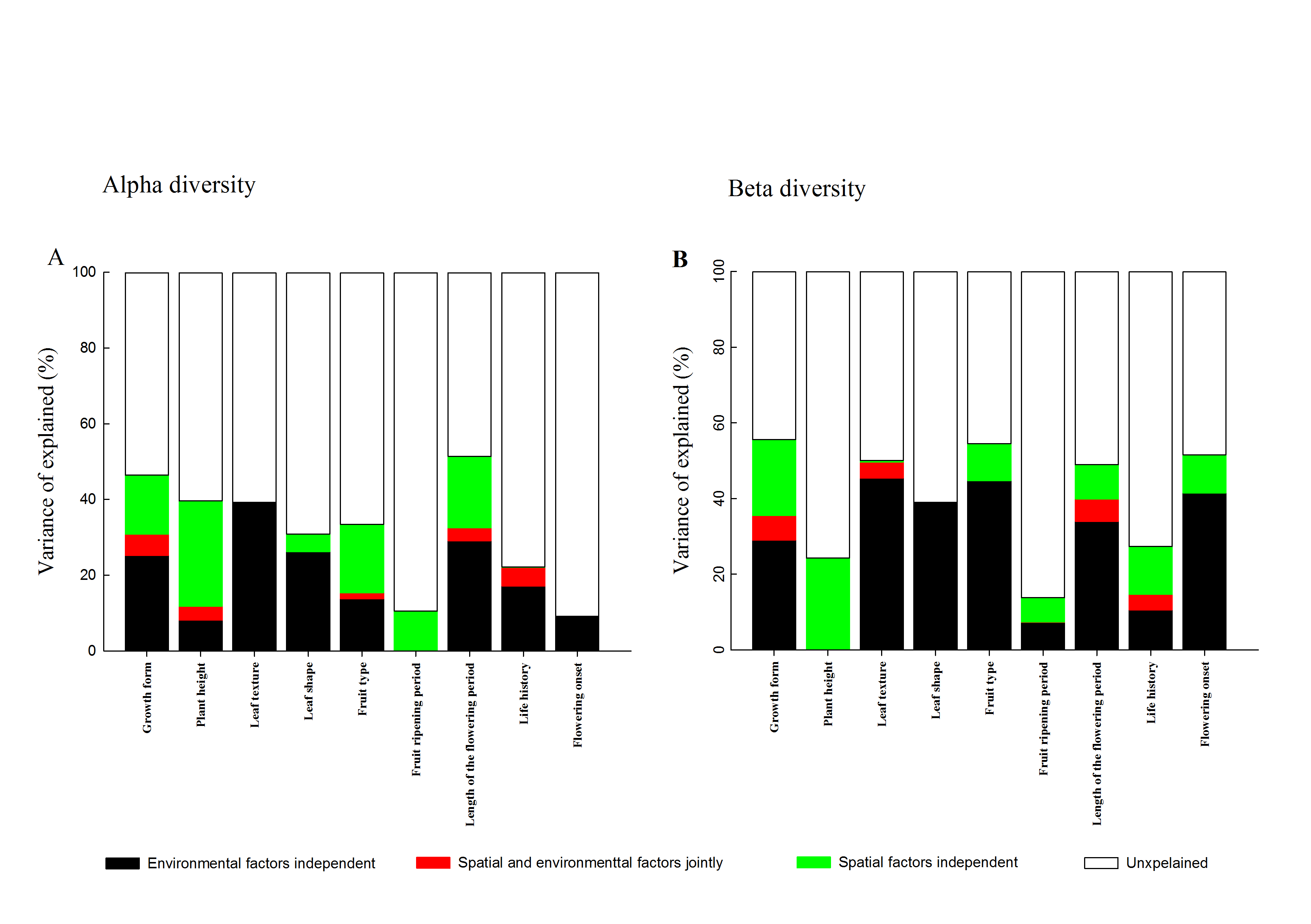

Supplement: Supplemental Information 6 — Notes: environmental factors independent, individual influence of environmental factors; spatial and environmental factors jointly, spared influence of spatial and environmental factors; spatial factors independent, individual influence of spatial factors; unexplained, the unexplained variation. [file peerj-07-6220-s006.png]

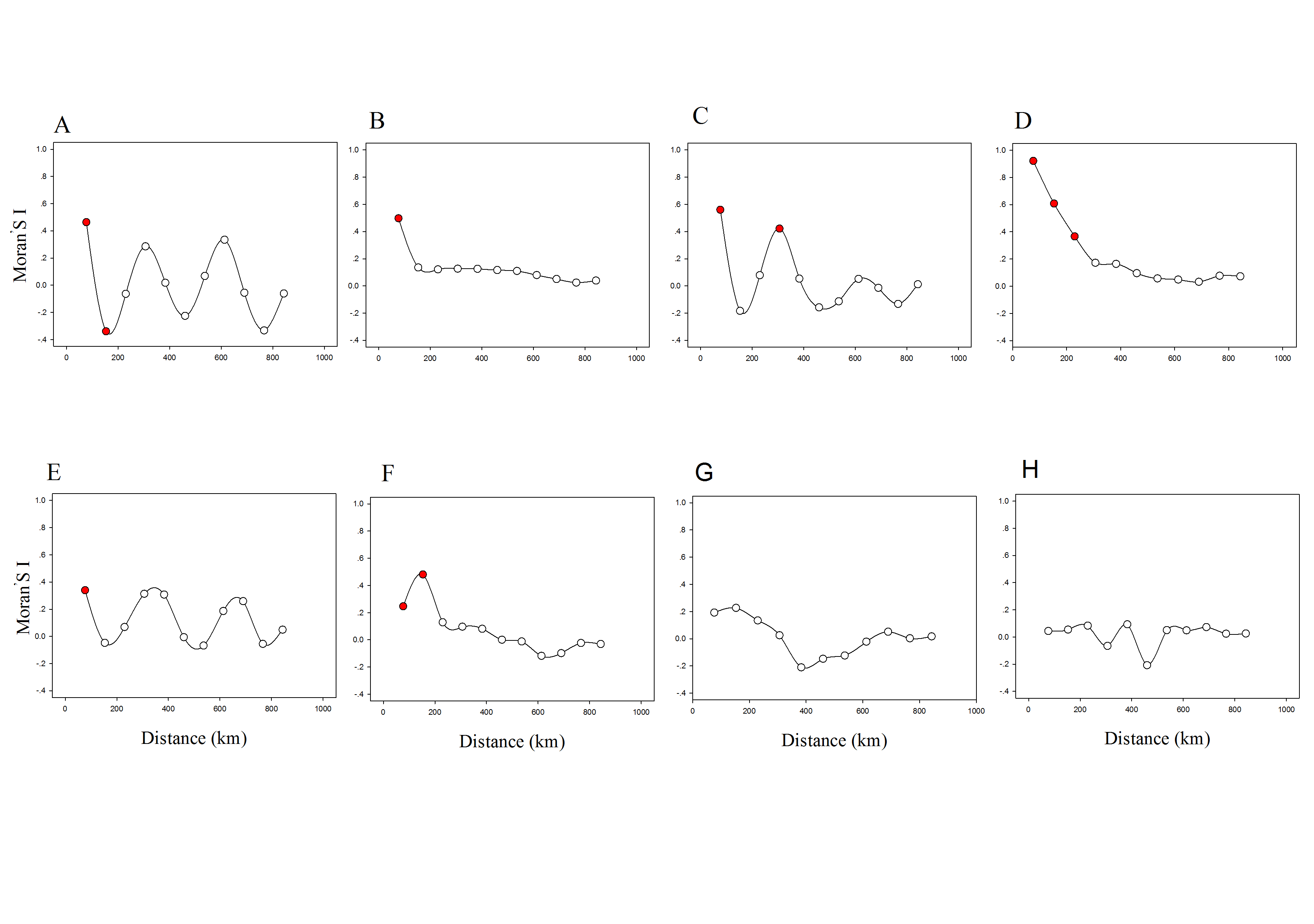

Supplement: Supplemental Information 7 [file peerj-07-6220-s007.png]
